# Redefining *Staphylococcus aureus* bacteremia: A structured approach guiding diagnostic and therapeutic management

**DOI:** 10.1016/j.jinf.2022.10.042

**Published:** 2022-11-09

**Authors:** Ilse J.E. Kouijzer, Vance G. Fowler, Jaap ten Oever

**Affiliations:** a Department of Internal Medicine and Radboud Center for Infectious Diseases, Radboudumc, Nijmegen, the Netherlands; b Department of Medicine, Duke University, Durham North Carolina, USA; c Duke Clinical Research Institute, Durham, North Carolina, USA

**Keywords:** Staphylococcus aureus bacteremia, Classification, Risk stratification, Diagnostic work-up, Individualized treatment

## Abstract

The current duration of therapy in patients with *Staphylococcus aureus* bacteremia (SAB) is based on differentiating complicated from uncomplicated disease. While this approach allows clinicians and investigators to group SAB patients into broadly similar clinical categories, it fails to account for the intrinsic heterogeneity of SAB. This is due in part to the fact that risk factors for metastatic infection and confirmed metastatic infection are considered as equivalent in most scoring systems. In this viewpoint, we propose a two-step system of categorizing patients with SAB. Initially, patients with SAB would be categorized as ‘high risk’ or ‘low risk’ for metastatic infection based upon an initial set of diagnostic procedures. In the second step, patients identified as ‘high-risk’ would undergo additional diagnostic evaluation. The results of this stepwise diagnostic evaluation would define a ‘final clinical diagnosis’ to inform an individualized final treatment plan.

## Introduction

*Staphylococcus aureus* bacteremia (SAB) is characterized by a unique ability to involve metastatic infections in almost every organ system in the body. Around 20% of the patients with SAB die within 30 days.^1^ In patients with SAB, the distinction between uncomplicated and complicated SAB is therapeutically important, as guidelines recommend different treatment durations for these two entities.^2,3^ However, this dichotomous classification strategy can incompletely reflect the heterogeneity of SAB. In this viewpoint, we discuss the limitations of the currently used definitions for SAB. We then propose a new approach to define the extent of infection in patients with SAB, providing an individualized framework for diagnosis and treatment.

## Current classification

There is currently no consensus on the exact definitions of ‘complicated’ and ‘uncomplicated’ SAB. In 1998, uncomplicated SAB was described in a study that investigated the association between adherence to consensus recommendations for treatment and patient outcome.^4^ In this study, SAB patients were classified into three categories: simple SAB, uncomplicated SAB, and complicated SAB, with differing durations of therapy with each category. Over a decade later, the Infectious Diseases Society of America (IDSA) guidelines for the treatment of methicillin resistant *S. aureus* (MRSA) infections omitted the category of ‘simple SAB’, and defined uncomplicated SAB as the presence of all of the following: a) negative follow-up blood cultures obtained 2–4 days after the initial set; b) defervescence within 72 h of initiating effective therapy; c) no prosthetic material; and d) no endocarditis and metastatic infection.^2^ These guidelines recommend that patients with uncomplicated SAB should be treated with at least 2 weeks of parenteral antibiotics.

The term ‘complicated’ SAB, further defined in 2003^5^ to include attributable mortality, metastatic foci or infection beyond the primary focus, or relapse, has become embedded in everyday practice and scientific research. Subsequent studies^6^ and the IDSA guidelines^2^ also included the presence of positive follow-up blood cultures in the definition of complicated SAB, because of its strong association with metastatic infection and death.^5,7^ Other investigators considered patient characteristics including persistent fever^5^, prosthetic material^2,4,8^, and hemodialysis dependence^9^ as indicative of complicated SAB.

## Shortcomings of the current classification

The major limitation of including predisposing host characteristics, features of bacteremia, and the clinical course (see below) in the definition of complicated SAB is that they increase the risk of metastatic infection and are not, in themselves, metastatic infection. As a result, it is possible for patients at risk for metastatic infection but without its confirmed presence to be diagnosed with and treated presumptively for complicated SAB.^2,3^ In addition, the current classification can discourage a precise clinical diagnosis, since SAB encompasses a much wider range of clinical manifestations than uncomplicated and complicated SAB. Explicitly defining patient characteristics and SAB diagnosis would allow a more personalized treatment including shorter durations of intravenous (IV) therapy and more convenient routes of administration.^10-14^

## Need for a new classification

**Fig. 1.**
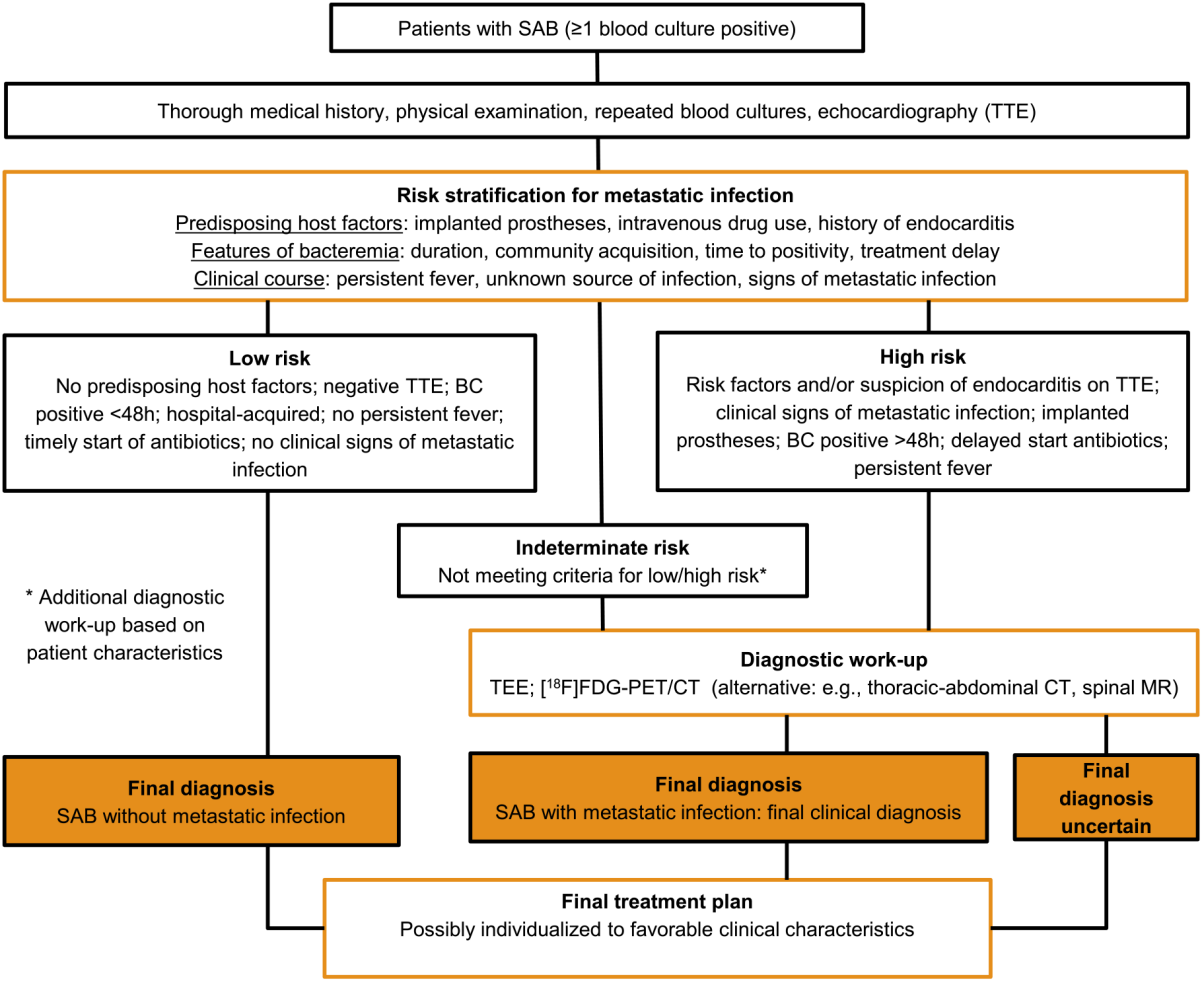
Proposal for a new approach for diagnosis and treatment in adults with *Staphylococcus aureus* bacteremia (SAB).

Clinicians need a classification for SAB that directs the diagnostic work-up and individualizes antibiotic treatment (Fig. 1). This framework can also be used to identify knowledge gaps for future research (Table 1). This proposed classification system begins after the initial positive blood culture with a requisite evaluation for every patient with SAB, including physical examination, repeat blood cultures, and echocardiography.^3^ Risk stratification based upon these results could then define patients at high or low risk of metastatic infection. Patients identified as high risk for complications could then undergo a more extensive diagnostic work-up to find or exclude these complications, in contrast to the patient without risk factors present at baseline or with negative results from the initial work-up (‘low-risk SAB’). Ideally, the result of the more in-depth diagnostic work-up would delineate the extent and nature of the patient’s *S. aureus* infection. This ‘final clinical diagnosis’, and not a designation of complicated SAB solely on the basis of the presence of factors that are associated with metastatic infections, would correspond with a certain treatment strategy for the average patients with this clinical picture, including route of administration, duration, and load reduction. The last step is to establish the final treatment plan for the individual patient by further streamlining or changing duration of the treatment based on clinical factors. Of course, for this classification to work in clinical decision making, it must be able to accurately identify the absence of metastatic infections in patients with SAB, even when traditional evaluations fail to identify one.

## Risk stratification to guide diagnostic work-up

### Factors associated with metastatic infection

A prerequisite for an individualized diagnostic approach is the ability to prospectively differentiate patients in whom metastatic infections are very unlikely from those with increased risk for these complications. Many studies have defined factors associated with mortality or metastatic infection in patients with SAB. These factors can be divided into 3 domains: predisposing host characteristics (prostheses, venous catheter, injection drug use, medical history of endocarditis), features of bacteremia (duration, short time to positivity, community acquisition, treatment delay), and the patient’s clinical course (persistent fever, unknown source of infection, and signs of metastatic infection).^2,5,7,15,16^ Besides these traditional factors to stratify patients at risk for metastatic infections, recent studies point towards the utility of inflammatory biomarkers for this purpose.^17^ And while the absence of these factors can help to identify patients with a low probability of metastatic infections^5,18^, it is insufficient to exclude them. For example, ~15% of a large cohort of SAB who exhibited none of the study-identified risk factors for complicated infection ultimately had this more serious outcome.^18^

### Stratifying the risk in clinical practice

While the use of risk factors present at baseline to identify patients prone to poor outcome in SAB is necessary, it is also insufficient. Thus, additional steps, including repeated physical examinations, follow-up blood cultures, and echocardiography, are essential to establish a patient’s risk for complications. Physical examination alone is fundamental in every patient with SAB but is insufficiently sensitive to exclude metastatic infection.^19,20^ For example, in a recent randomized controlled trial (RCT) on the management of staphylococcal bacteremia, mandatory use of follow-up blood cultures and echocardiography reclassified a significant proportion of SAB patients from uncomplicated to complicated infection.^21^ Careful risk factor stratification may ultimately be shown to promote diagnostic stewardship by limiting testing in low-risk patients while supporting more extensive evaluation in patients at high-risk for complications. Identification of risk factors, alone or in combination, that exclude underlying infectious complications and render additional diagnostic testing unnecessary is therefore an important research question (Table 1).

Recent reports suggest that certain clinical factors are already used in this manner to justify ordering echocardiography^22^ or other imaging^23^ in patients with SAB. This use of risk stratification is not an absolute classification in SAB, but rather should be considered as supplemental data with which to assess the individual risk of a particular patient with SAB. Using our proposed risk stratification strategy (Fig. 1), a significant minority of SAB patients will be indeterminate for either low-risk or high-risk SAB, such as patients in whom follow-up blood cultures are not properly collected or when it is unclear what risk should be assigned to certain prosthetic material. These indeterminate patients need an additional diagnostic evaluation based on specific patient characteristics. In the eventuality that this diagnostic work-up fails to exclude elevated risk, these patients must be regarded by default as high-risk SAB for decisions involving treatment. Thus, a future research priority should be to identify the requisite diagnostic studies needed to determine safe, effective treatments for patients with SAB.

## Detection of metastatic infectious foci

### Echocardiography

Using a risk stratification for diagnostic investigations is only helpful if there are diagnostic modalities that can reliably detect metastatic infections. For either diagnosing or excluding endocarditis in SAB, standard clinical practice is to perform echocardiography in patients with SAB^2,3^, as a significant portion of patients with SAB have endocarditis in absence of clinical signs.^20,24,25^ Scoring systems have been developed to determine the necessity of (transesophageal) echocardiography.^26,27^ Transesophageal echocardiography (TEE) should be performed with low threshold in patients with negative TTE but persistent suspicion of endocarditis and in patients with prosthetic heart valves and/or cardiac implantable electronic devices.^28^ On the other hand, the combined absence of certain risk factors and prognostically unfavorable features of bacteremia can probably obviate the need of TEE.^26^ Future research should focus on how clinical prediction scores can be improved to reduce the number of patients classified as high risk for endocarditis – and thus requiring TEE – while maintaining acceptable negative predictive value.^26^ It must be said, however, that such trials will be difficult to conduct as the event rates are low in the very low risk patient group and will therefore require large numbers to demonstrate any difference between the strategies.

Another question is whether there are subgroups of patients with SAB in whom echocardiography can be safely omitted altogether. For example, patients with a very low *a priori* risk of endocarditis, or patients with transient bacteremia already receiving extended courses of parenteral antibiotics for other sites of infection might not always require TTE. Scenario-based research has shown that clinicians already actively engage in risk stratification when applying echocardiography strategies.^22^ It is important to note, however, that TTE is widely available, safe, inexpensive, and standard of care for patients with SAB.^3^ Thus, any decision to withhold echocardiography from SAB patients will need to be shown not to cause harm in high quality, adequately powered, and broadly generalizable trials.

### [^18^F]FDG-PET/CT to detect metastatic infection

Metastatic infections other than endocarditis are common in SAB, but may be clinically occult in up to 70% of patients.^5,23,29,30^ A promising recent diagnostic modality in patients with SAB is 2-[^18^F]fluoro-2-deoxy-d-glucose positron emission tomography with combined computed tomography ([^18^F]FDG-PET/CT). Nonrandomized studies have shown that the incorporation of [^18^F]FDG-PET/CT into the diagnostic work-up of high-risk SAB patients is associated with lower mortality and relapse rates.^23,30,31^ The high sensitivity of [^18^F]FDG-PET/CT for extracardiac infections enables a shorter treatment duration in high-risk patients if [^18^F]FDG-PET/CT and echocardiography are negative.^32^ In addition to the detection of extracardiac metastatic infection, [^18^F]FDG-PET/CT has a recognized role in Europe in the work-up of prosthetic valve endocarditis.^28^ Unfortunately, [^18^F]FDG-PET/CT is not universally available and in the postoperative setting it could be difficult to distinguish physiologic post-surgical [^18^F]FDG uptake from infection.^33^ Thus, RCTs will be required to determine the optimal role of [^18^F]FDG-PET/CT in SAB management (Table 1).^34^ A recent Dutch retrospective study assessed the impact of [^18^F]FDG-PET/CT on treatment adjustment in SAB patients that already had a treatment indication of at least 6 weeks before performing the nuclear imaging because of complications present.^35^ [^18^F]FDG-PET/CT results led to treatment modification only in those patients with clinically suspected endovascular infection and could be safely omitted in other patients who were clinically stable. In a recent prospective observational Israeli study treatment modifications commonly occurred following [^18^F]FDG-PET/CT and the results suggested that the benefit may have been greater in low-risk patients than high-risk patients.^31^ Another observational study among 91 patients (including 39 due to *S. aureus*) with late acute hematogenous PJI, showed that the risk of concomitant PJI in an additional asymptomatic arthroplasty was very low^36^, findings that were recently confirmed.^37^

## Establishing a final treatment plan

After the risk-adapted diagnostic work-up, the final clinical diagnosis describes the metastatic infections and informs the definitive treatment plan. Importantly, an accurate final clinical diagnosis is not always possible, or clinical suspicion for an underlying metastatic infections is high even in the absence of an established diagnosis. In both such settings, patients should be treated for complicated SAB, with extended courses of antibiotics.

In SAB patients, with or without metastatic infection, a more individualized treatment might eventually be possible. The results of the SABATO randomized trial were recently presented and showed non-inferiority of oral switch therapy after 5–7 days of IV treatment compared to a complete 14-day IV course in the small group of patients at low risk of complications.^38^ A recent observational study suggested that a minority of patients with SAB can be treated with an abbreviated course of antibiotics.^39^ Partial oral treatment was shown to be non-inferior to a complete IV treatment course in stable patients with left-sided Gram-positive infective endocarditis and a satisfactory clinical response to initial treatment, although only 47 of orally treated patients had SAB and MRSA was excluded in this randomized controlled trial.^40^ In a retrospective single center study, oral step-down was associated with low mortality and absence of relapse in selected patients with metastatic infection in whom endovascular infection had been ruled out with echocardiography and [^18^F]FDG-PET/CT.^12^

Future randomized clinical trials will further establish the pre-requisites for less intensive and personalized treatment in SAB patients. The SAB7 randomized clinical trial will assess whether 7 days of antibiotic treatment in patients with uncomplicated SAB, defined as rapid defervescence and clearance of bacteremia in the absence of endocarditis and other risk factors for metastatic infection, is non-inferior to 14 days of treatment.^41^ Currently, a randomized controlled trial investigates non-inferiority of oral switch of antibiotic treatment as compared with entirely IV antibiotic treatment in patients with left-sided endocarditis caused by multi-susceptible staphylococci.^42^ The SAFE-trial in the Netherlands will determine whether patients with metastatic infections including endocarditis with favorable characteristics can be safely treated for 4 weeks instead of 6 weeks.^43^ The International (SNAP) Staphylococcus aureus Network Adaptive (SNAP) trial has begun enrolling patients and is evaluating, among other things, early oral switch for both uncomplicated and complicated infections.^44^

## Biomarkers to individualize treatment duration

A possible way to personalize treatment even more could be with the use of biomarkers, as is already done with procalcitonin in intensive care unit and in patients with community-acquired pneumonia. If shown to be effective in clinical studies, one potential example of personalizing antibiotic therapy based upon novel diagnostic platforms includes the use of microbial cell-free *S. aureus* DNA from plasma to define the duration of antibiotic therapy in patients with SAB by identifying when *S. aureus* DNA drops below limits of detection.^45^

## Follow-up

Structured follow-up is also essential after discharge. First, clinical response should be monitored to detect clinical failure. Second, patients are increasingly completing treatment at home, either through oral therapy^12,40^ or outpatient parenteral antimicrobial therapy (OPAT)^46^ and, regardless of the route of administration, treatment-related toxicities should be actively monitored. Based on the above, it is sometimes necessary to adjust the treatment plan. Finally, follow-up must continue well beyond the end of treatment as up to 5% of patients will experience a relapse.^47^

## Conclusion

The framework proposed here for the classification and management of patients with SAB has the potential to solve the shortcomings of the current simplistic definition of uncomplicated and complicated SAB. It consists of four steps: (1) risk stratification for the presence of metastatic infection that (2) directs a diagnostic work-up in search for these infections leading to (3) ‘final clinical diagnoses’ corresponding with a general direction for treatment that (4) can be individualized to favorable clinical characteristics. This framework provides guidance to the clinician and a context for future research to improve patient outcome and individualized treatment.

## Figures and Tables

**Table 1:** Future research questions concerning diagnosis and treatment in SAB.

1. The absence of which risk factors and negative results of an initial work-up makes additional diagnostic investigations for metastatic infections including infective endocarditis unnecessary? 2. Which novel predictive factors can improve the discriminatory power of clinical risk stratification? 3. Is a risk-based diagnostic work-up associated with improved outcomes and is it cost-effective? 4. How can clinical prediction scores be improved to reduce the number of patients classified as high risk for endocarditis while maintaining acceptable negative predictive value? 5. Which high-risk patients need an [^18^F]FDG-PET/CT? 6. Which patients can be treated with oral antibiotics and/or for a shorter duration? 7. Which biomarkers are useful for individualizing treatment duration?
